# Supplementary material for: Healthy Food Environments in Early Learning Services: An Analysis of Manager Survey Responses, Menus and Policies in Regional New Zealand Early Childhood Education and Care Centres
Source: Int J Environ Res Public Health. 2022 Apr 13;19(8):4709. doi: 10.3390/ijerph19084709 (PMC9028152; doi:10.3390/ijerph19084709)
Supplement: Supplementary file 1 [file ijerph-19-04709-s001.zip › ijerph-1636046-Supplementary.pdf]

Supplementary Table S1. Inter-rater agreement on the 10 indicators of Shortened WellCCAT-NZ Policy Analysis tool

**ELS Policies (n=14)**							
**Nutrition Education**		**Nutrition Standards**		**Promotion of healthy environment**		**Communication**	
Indicator	% agreement	Indicator	% agreement	Indicator	% agreement	Indicator	% agreement
NE1	7/14 – 50%	NS1	8/14 – 57%	NP1	11/14 – 79%	CE1	14/14 – 100%
NE2	11/14 – 79%	NS2	13/14 – 93%	NP2	11/14 – 79%	CE2	13/14 – 93%
		NS3	10/14 – 71%			CE3	11/14 – 79%

Supplementary S1

Nourishing Hawke's Bay food environments survey of Early Learning Services

ELS Survey

Welcome

1. **Consent Form**

Please confirm that you

- have read the **Participant Information Sheet**
- have understood the nature of the research and why my ELS has been invited.
- have had the opportunity to ask questions and have them answered to your satisfaction.
- have had sufficient time to think about participation and to talk about it with the person(s) of your choice.
- agree for your ELS to take part in this research and complete a self-review online questionnaire on ELS food environments (approximately 7-10 minutes)
- understand that participation is voluntary
- understand that you are free to withdraw participation at any time without giving a reason
- understand that the researchers guarantee confidentiality of your identity, and that ELS identification will be retained for feedback and support purposes as outlined in the Participant Information Sheet
- understand you will receive a letter with the results of the project at the end of study
- understand that this consent form will be kept for 6 years, after which it will be destroyed

Do you agree to participate?
*

○ Yes

○ No

Policies and Programmes

2. **Does your centre have any written policies, procedures or guidelines for staff and/or parents around drinks/beverages?**

This might include messaging around water only / water as the best drink / water and unflavoured milk only. It may include drinks that are provided by the centre, drinks brought from home, and drinks consumed on outings or at events.

- Yes
- No
- Don't know

3. **Does your centre have any written policies, procedures or guidelines for staff and/or parents about food and/or nutrition?**

These might include guidelines about lunchbox contents or practices at meal times, foods provided by the centre and/or brought from home, etc.

- Yes
- No
- Don't know

4. **Does your centre <u>currently</u> participate in any of these food and nutrition programmes?**
(tick all that apply)

- ☐ 5+ A Day (Fresh Fruit and Vegetable Charitable Trust)
- ☐ Healthy Heart Award (Heart Foundation)
- ☐ KidsCan Charitable Trust
- ☐ Enviroschools / Te Aho Tu Ra
- ☐ Garden to Table
- ☐ Other programme. Please specify:
- ☐ No, we do not participate in a nutrition programme

Food Provision at your ELS

5. **Which of the following best describes the daily food provision for children/tamariki at your centre:**

- ○ The centre provides all (meals and snacks) food for children/tamariki to eat
- ○ The centre provides some meals/snacks
- ○ All food is provided by the home (parents/whānau)

6. **Which of these meals and/or snacks does your centre provide the food for children/tamariki to eat?**
(tick all that apply)

- ☐ Breakfast
- ☐ Morning snack
- ☐ Lunch
- ☐ Afternoon snack
- ☐ Late (pre-dinner) snack
- ☐ Dinner

Special Occasions

7. **Do children/tamariki bring food from home to your centre for special occasions, such as a themed day, celebrations or birthday parties?**

- ○ No, it is not permitted
- ○ Yes, for some special occasions
- ○ Yes, for most or all special occasions

8. **Please use the sliding scale to evaluate overall the healthiness of foods and/or beverages brought from home for special occasions:**

	0%	50%	100%
**Green Items** (healthy Items, classified as 'everyday') fruits, vegetables, plain milk, bread, grains/cereals, eggs, beans and similar sources of protein			
**Red Items** (unhealthy Items, classified as 'occasional') muffins, scones, pizza, sausage rolls, nuggets, cake, sweets, flavoured milk, soft drinks and fish and chips)			

9. **Does your centre provide food and/or beverages for special occasions such as themed day, celebrations like birthday parties, or events?**

- Yes
- No

10. **Please use the sliding scale to evaluate overall the healthiness of foods and/or beverages your centre provides for special occasions:**

	0%	50%	100%
**Green Items** (healthy Items, classified as 'everyday') fruits, vegetables, plain milk, bread, grains/cereals, eggs, beans and similar sources of protein			
**Red Items** (unhealthy Items, classified as 'occasional') muffins, scones, pizza, sausage rolls, nuggets, cake, sweets, flavoured milk, soft drinks and fish and chips)			

Fundraising

11. **Does your centre use food and/or beverages in fundraising?**

- ○ Yes
- ○ No

12. **Please use the sliding scale to evaluate overall the healthiness of foods and/or beverages used for fundraising:**

	0%	50%	100%
**Green Items** (healthy Items, classified as 'everyday') fruits, vegetables, plain milk, bread, grains/cereals, eggs, beans and similar sources of protein			
**Red Items** (unhealthy Items, classified as 'occasional') muffins, scones, pizza, sausage rolls, nuggets, cake, sweets, flavoured milk, soft drinks and fish and chips)			

13. **How often does your centre carry out fundraising activities with food and/or beverages?**

- Weekly
- Once every 2-3 months
- Every six months
- Annually

ELS Culture and kaupapa

14. **How would you describe the culture/kaupapa around healthy eating at your centre?**

**Very strong** *(policies in place, strong healthy food practices, staff and parents strongly support kaupapa of healthy food)*	**Strong**	**Medium** *(some policies and practices support healthy food, mixed support from staff and parents for the kaupapa of healthy food)*	**Weak**	**Very weak** *(no policy, considerable unhealthy foods provided, healthy eating is a low priority for staff and parents)*
○	○	○	○	○

15. **Please rate the following statements:**

	Rating				
	Strongly Agree	Agree	Neither agree nor disagree	Disagree	Strongly Disagree
**Nutrition and healthy eating are highly prioritized at our centre** *(incorporated into service policy/vision/goals)*	○	○	○	○	○
**Nutrition training is provided and regularly updated for all staff** *(including cooks and food service staff)*	○	○	○	○	○
**Staff consistently act as role models for healthy eating** *(teachers sit down and eat with tamariki, and eat healthy food in front of children)*	○	○	○	○	○
**We frequently communicate with parents and whānau about nutrition and healthy eating** *(e.g. through enrolment information, newsletters, website/Facebook, posters, app)*	○	○	○	○	○
**Centre management and staff share a strong collective vision around hauora/health**	○	○	○	○	○
**Our centre and parents/whānau share a strong collective vision around hauora/health**	○	○	○	○	○

16. **Does your centre experience any of these barriers to providing or promoting healthy food to children/tamariki?**
(tick all that apply)

- ☐ Lack of support from administration or management
- ☐ Lack of staff training on nutrition education
- ☐ Lack of training for cook / food service staff
- ☐ Lack of support from teachers and/or staff
- ☐ Lack of support from parents/ whānau
- ☐ Insufficient funds
- ☐ Concerns about food intolerances or allergies
- ☐ Concerns about food-related choking
- ☐ Requirements around food safety
- ☐ Other, please specify
- ☐ None of the above

General Questions

17. **What is your ELS Ministry of Education Service ID?**

(We will use this ID to link to the Early Learning Services Database so we can see how many children are enrolled in your service, and the type of service you are registered as. You will not be individually identified in any of the reporting or results)

18. **Which of these categories best describes your position within your centre?**

- Centre Manager / Director / Head Teacher / Kaiwhakahaere
- Teacher / Kaiako
- Cook / Food service staff
- Board member / Trustee
- Administrator / Support Staff
- Parent / Whanau (or unpaid volunteer)
- Other, please specify:

19. **Please attach electronic documents (PDF, Word, jpeg/photo) of any written policies, procedures or guidelines for staff and/or parents related to food and nutrition.**

If you prefer you can email us at: nourishinghb@eit.ac.nz. Please include your MoE ID.

Browse...

20. **If you provide food daily to children, please attach an electronic copy of your menu or food record for the last week (5 days).**

If you prefer, you can email us at: nourishinghb@eit.ac.nz. Please include your MoE ID.

Browse...

21. **What might enable you to create a healthier food environment in your centre?**

(*For example: Any specific actions or support that may increase the provision of healthier foods and/or decrease provision of unhealthier foods, etc.)*

Supplementary S2 - Shortened Wellness Child Care Assessment Tool modified for use in NZ (WellCCAT-NZ) Score Sheet

Centre name: **Date of Assessment:**

The *Shortened WellCCAT-NZ Score Sheet* summarizes a centre's food and nutrition policy scores based on four sections containing a total of 10 policy items. Each item is rated as "0" (not mentioned), "1" (weak statement regarding the item), "2" (strong statement regarding the item)

Comprehensiveness (score out of 10) is calculated by counting the number of items rated as "1" or "2". **Strength** (score out of 10) is calculated by counting the number of items rated as "2." 'Nutrition Standards' refers to the Ministry of Health's Healthy Food and Drink Guidance (2020).

	Nutrition Education	Rating
NE1	**Addresses the inclusion of nutrition education for children**	
NE2	**Addresses the provision of nutrition education training for teachers and those involved in cooking/food preparation.**	

	Nutrition Standards for Food and Beverages Provided	Rating
NS1	**Addresses the implementation of the Nutrition Standards for foods and beverages provided to children or food provision from home (e.g. centre provides some/all foods and/or parent provide some/all foods)**	
NS2	**Addresses implementation of the Nutrition Standards for rewards, celebrations, events, and/or special occasions, and fundraising activities.**	
NS3	**States that beverage provision is milk and water only (no sugary drinks at any time).**	

	Promoting a Healthy Food and Nutrition Environment	Rating
NP1	**Encourages teachers to be role models for healthy eating (eg. sitting with children during meals, assisting children to gauge fullness) including staff consumption of foods and/or beverages meeting the Nutrition Standards.**	
NP2	**Addresses specific course of action when food from home does not meet nutritional standards**	

	Food and nutrition communication and evaluation	Rating
CE1	**Addresses the communication of the centre food and nutrition policy to students, staff and parents.**	
CE2	**Addresses the provision of nutrition information for parents.**	
CE3	**Specifies a suitable timeframe for revising and updating the centre food and nutrition policy.**	

Supplementary S2 - Shortened Wellness Child Care Assessment Tool modified for use in NZ (WellCCAT-NZ) Score Sheet

Centre name: **Date of Assessment:**

[redacted]

Total Comprehensiveness score /10

Total Strength score /10

How to Rate School Food and Nutrition Policy Statements

ELS Wellness policies are evaluated based on the degree to which they address 10 policy items, which are categorised into four sections. The sections include Nutrition Education, Nutrition Standards for Foods and Beverages, Promoting a Healthy Food and Nutrition Environment, and Communication and Evaluation.

For each of the 40 policy items, policy statements are to be rated "0," "1," or "2," using the definitions below. Three items may be rated "NA" if the policy statement does not apply. This evaluation tool lists each policy item followed by an explanation of the item and examples of "1" and "2" statements.

		**Explanation**
**0**	**= Not Mentioned**	The item is not addressed in the text of the policy.
**1**	**= Weak Statement**	Assign a rating of "1" when the item is mentioned, <u>*but*</u><br>• The policy will be hard to enforce because the statement is *vague*, *unclear*, or *confusing.*<br>• Statements are listed as *goals*, *aspirations*, *suggestions, objectives,* or *recommendations.*<br>• There are *loopholes* in the policy that weaken enforcement of the item, such as a *disclaimer* that states that policies are non-enforceable and/or subject to change without notification.<br>• The policy mentions a *future plan to act* without specifying when the plan will be established.<br>Words often used include*:* ***may, can, could, should, might, encourage, suggest, urge, some, partial, make an effort,*** and ***try***.
**2**	**= Meets / Exceeds Expectations**	Assign a rating of "2" when the item is mentioned, and it is clear that the child care center is committed to making the item happen because:<br>• The item is described using specific language (e.g., a concept followed by concrete plans or strategies for implementation).<br>• Strong language is used to indicate that action or regulation is required, including: ***shall, will, must, have to, insist, require, all, total, comply*** and ***enforce.***<br>• A centre is unable to enforce an item, but the goal is clearly stated (e.g., "teachers eat the same foods served to children").<br>• Statements in parent handbooks that are preceded with "please" (e.g., "please do not bring in sweets or dessert foods for your child's lunch") are acceptable for a rating of a "2."<br>• Instructional statements in the staff manual (e.g., "do not use food as reward or punishment") are acceptable for a rating of a "2."

Evaluating hint One method for deciding between a rating of a "1" and a "2" is to consider the scenario of a parent approaching an ELS' centre manager to discuss an issue. If the policy is ambiguous on how the ELS should handle the issue at hand, rate the item as a "1." If the

written policy gives clear guidance about how to decide whether the ELS complies with the policy, rate the item as a "2."

Section A. Nutrition Education

#	Item	Rating	Rating Guidance
NE1	**Addresses the inclusion of <u>nutrition education</u> for children**		**Note:** This item does not include informal nutrition education during mealtimes.
		0	Not mentioned.
		1	**Either of the following:**<br>• Describes a general health and wellbeing curriculum or lesson plan, and implies but does not ensure, that lessons address nutrition<br>• Suggestions that children receive nutrition education<br>**Examples:**<br>• *"Our educational program is designed to promote sound nutritional practices"*<br>• *"Activities to promote good nutrition are planned by the staff throughout the year"*<br>• *"Educational experiences will assure that preschool children will recognize and eat a variety of nutritious foods"*
		2	**Requires that n**utrition education is provided.<br>**Examples:**<br>• *"The centre shall implement a health education curriculum that shall include lessons on nutrition, fitness, hand-washing…"*<br>• *"Our preschool curriculum includes lessons on nutrition"*
NE2	**Addresses the provision of <u>nutrition education training for teachers and those involved in cooking/food preparation</u>.**	0	Not mentioned.
		1	**Either of the following:**<br>• Provision of nutrition education/food preparation training is suggested.<br>• Nutrition education/food preparation training is provided to staff, but the exact type is not specified<br>**Example:** *"Teachers will be trained in health education, which includes training on topics <u>such as</u> nutrition…."*
		2	**Requires**<br>• Provision of nutrition education which can include training in facilitating or planning nutrition-related activities<br>• Could also include gaining food preparation certification<br>**Example:** *"Teachers participate in annual training on nutrition education activities for the children…"*<br>*"Staff involved in food preparation will be trained by a registered dietician in menu planning annually"*<br>*"Food service staff will receive training in procedures and techniques to support children's nutritional needs."*

Section B. Nutrition Standards for Food and Beverages Provided

Supplementary S2 - Shortened Wellness Child Care Assessment Tool modified for use in NZ (WellCCAT-NZ) Score Sheet

Centre name: **Date of Assessment:**

<table>
<tr><th>#</th><th>Item</th><th colspan="2">Rating Guidance</th></tr>
<tr><td rowspan="3">NS1</td><td rowspan="3">Addresses the implementation of the Nutrition Standards for foods and beverages <u>provided to children or food provision from home</u> (e.g. centre provides some/all foods and/or parent provide some/all foods)</td><td>0</td><td>Not mentioned.</td></tr>
<tr><td>1</td><td>Any of the following:
Vague and/or suggested
Mentions compliance to one or more standards but does not specify how it will do so (no specific criteria state)
Example: “All foods comply with the Ministry of Health’s Food and Nutrition Guidelines”
“We follow the Ministry of Health’s guidelines”

“We encourage you to pack healthy meals for your child and to avoid packing foods like lollies or chips”
“When packing lunch, limit snack foods and desserts”</td></tr>
<tr><td>2</td><td>Requires compliance to nutrition standards <u>and</u> that specific criteria is mentioned

Provides parents with written nutrition standards for foods brought into the centre and requires parents to follow the standards (even if you feel standards are weak)

Examples:
“All foods served shall meet the following standards…” (eg. Whole fruit is served at all meals, at least ½ of breads and cereals are whole grain, all foods are low fat, etc.)

“Foods including fizzy drinks, fruit drinks, flavoured milk, lollies, chocolate, sugary cereals, and/or hot chips are not allowed to be served or consumed in our ELS centre”

“Parents must pack lunch and/or snack that includes fruits and/or vegetables and whole grain breads and excludes lollies, fizzy drinks and dessert foods”

“No lollies, fizzy drinks, energy drinks, chocolate, or other sweets are allowed to be brought into the centre”</td></tr>
</table>

<table>
<tr><td rowspan="2">NS2</td><td rowspan="2">Addresses implementation of the Nutrition Standards for <u>rewards, celebrations, events, and/or special occasions, and fundraising activities.</u></td><td>NA</td><td>The policy specifies that the centre does not conduct fundraising activities</td></tr>
<tr><td>0</td><td>Either of the following:
Not mentioned.
Policy allows traditional party foods (e.g., cake, confectionery, or biscuits) without stating any limits
The following are insufficient for a rating of “1” or “2”
Requiring parents to notify the centre before bringing in foods
Outside of the context of celebrations, stating that no food from home is permitted <u>without</u> specifying “at all times”</td></tr>
</table>

Supplementary S2 - Shortened Wellness Child Care Assessment Tool modified for use in NZ (WellCCAT-NZ) Score Sheet

Centre name: **Date of Assessment:**

			Does not mention nutrition standards for food sold for fundraising, or mentions the use of unhealthy food for fundraising<br>**Example**: "*The fundraising activities may include sausage sizzles, confectionary sales, bake sales, and special events*"
		1	**Any of the following:**<br>• Vague, suggested, or weakened by exceptions such as staff discretion.<br>• Limits the number of birthday celebrations with traditional party foods to no more than 1 per month<br>• Limits some traditional party foods and beverages but specifically allows others<br>• Suggests or recommends the use of non-food alternatives for celebrations and classroom rewards<br>• The centre provides foods for celebrations and it is stated or implied that the food will be "healthy"<br>• Discourages food as a reward<br>• Only allows healthy food as a reward<br>• Regulations of food sold for fundraising are vague, suggested, or weakened by exceptions such as staff discretion<br>• Regulations of food sold for fundraising only apply to a limited group of foods or often to food sales on centre grounds<br>**Examples**: *"With the help of a dietician, we have created a menu from which staff may select items for celebrations"*<br>*"Some ideas for celebrations are birth crowns, place mats or singing 'Happy Birthday'"*<br>*"There will be no sausage sizzles or food sales for fundraising allowed on centre grounds."*
		2	Requires regulation or prohibition of ALL foods and beverages available during on-site rewards, celebrations and fundraising or "at all times" (or an equivalent, such as "at any time" or "no matter the occasion"). For a "2", regulations must meet o**ne of the following criteria:**<br>• Define specific nutrition standards for all foods or beverages available to be used as rewards, for fundraising and during celebrations or brought from home for celebrations (eg. Maximum calorie, sodium, sugar, and/or saturated fat content of ALL items)<br>• Provide a specific and restricted list of healthy food and beverage items allowed for fundraising, rewards and at celebrations or at all times (eg. Limiting to fruits and whole grain cereals)<br>• Provide a comprehensive list of <u>prohibited</u> traditional party/fundraising foods and beverages (eg. Baked goods, sweetened beverages, and confectionary) at celebrations and fundraising, or at all times<br>• Food at centre celebrations and/or rewards are prohibited<br>**Examples:**<br>*"Parties at our centre are not celebrated with food. Instead teachers will choose a special activity to celebrate each child's birthday"*<br>*"All birthday treats must be healthy snacks or fruits. No ice cream, cakes, biscuits, lollies or cupcakes are allowed"*<br>*"Fundraising activities will be conducted using non-food alternatives only"*<br>*"Food is never to be used as a punishment or reward"*

Supplementary S2 - Shortened Wellness Child Care Assessment Tool modified for use in NZ (WellCCAT-NZ) Score Sheet

Centre name: **Date of Assessment:**

#	Item	Rating	Guidance
NS3	**States that beverage <u>provision</u> is <u>milk and water only</u> (no sugary drinks at any time).**	0	Not mentioned.
		1	Any of the following:<br>• Vague and/or suggested<br>• Discouraged or limited without prohibiting<br><br>**Example** *"Fizzy drinks are discouraged from being consumed at our centre"*
		2	Requires prohibition and regulation.<br>• Provision and/or sale of sugar-sweetened beverages is not allowed at our centre<br>• Parents are not allowed to supply sugar-sweetened beverages from home<br>**Example:** *"Our ELS has a strict policy on the consumption of sugar-sweetened beverages. These items will neither be provided and must not be brought from home"*

Section 3. Promoting a Healthy Food and Nutrition Environment

#	Item	Rating	Guidance
NP1	**Encourages <u>teachers to be role models for healthy eating</u> (eg. sitting with children during meals, assisting children to gauge fullness) including <u>staff consumption of foods and/or beverages meeting the Nutrition Standards</u>.**	0	Not mentioned.
		1	**Either of the following:**<br>• Vague and/or suggested.<br>• Mentions teachers modeling healthy eating habits but does not explicitly require them to eat the same foods offered to children<br><br>**Example:**<br>*"Teachers are encouraged to adhere by the school nutritional standards"*<br>*"Teachers should listen to children's hunger cues"*
		2	**Requires:**<br>• Staff to model healthy eating behaviours during all interactions with students<br>• Staff to comply with the centres (or standards such as NS1) prescribed nutritional standard<br><br>**Example**:<br>*"Teachers must adhere to the same nutrition standards prescribed for children"*<br>*"Staff are required to eat with children during meals"*<br>*"Periodically remind children that when their bellies feel full, they should stop eating"*

#	Item	Rating	Guidance
		NA	No food from home is permitted, and there are no policy statements addressing this item
		0	Not mentioned

Supplementary S2 - Shortened Wellness Child Care Assessment Tool modified for use in NZ (WellCCAT-NZ) Score Sheet

Centre name: **Date of Assessment:**

NP2	**Addresses specific <u>course of action</u> when <u>food from home</u> does not meet nutritional standards**	1	**Any of the following:**<br>• Vague and/or suggested.<br>• Staff monitor food from home, but when foods or beverages are not in compliance with nutritional standards, no course of action is specified<br>• Staff monitor food from home for food safety, allergens or choking hazards only
		2	Staff are required to take a specific course of action when food from home does not meet centre's nutritional standards<br>**Examples:** • *"If parent consistently sends foods that does not meet nutritional requirements, the Centre Manager will discuss this with the parent(s) and refer to a dietician if appropriate"*<br>• *"If children bring prohibited foods, these foods will be returned home with a copy of our nutrition standards"*

Section D. Food and Nutrition Communication and Evaluation

CE1	**Addresses the <u>communication</u> of the centre food and nutrition policy to students, staff and parents.**	0	Not mentioned.
		1	**Either of the following:**<br>• Vague and/or suggested.<br>• Mentions addressing the communication/accessibility of the food and nutrition policy but does not specify the method through which this takes place<br>• Communication is required, but the person or group responsible is vague<br>• Specifies who is responsible, but communication is only suggested
		2	Designates a specific individual or group responsible for communication of the nutrition policy<br>**Example:** *"Parents will be notified of the centre food and nutrition policy via email during enrollment"*<br>*"Staff and parents will be notified via email and the newsletter when policies are modified, reviewed and updated"*

CE2	**Addresses the provision of <u>nutrition information</u> for <u>parents</u>.**	0	Not mentioned.
		1	**Either of the following:**<br>• Vague and/or suggested.<br>• Describes the provision of nutrition education for parents but does not mention specific methods or activities.<br>• Lists nutrition-related services in policy, handbook or similar
		2	• Provision of nutrition education for parents is required and at least one potential method of educating parents is mentioned (eg. Provision of workshops on nutrition or providing nutrition newsletters)<br>• Nutrition education materials are included in the parent handbook<br>• Ensures that parents are provided with referrals for health- and/or nutrition- related services where appropriate

Supplementary S2 - Shortened Wellness Child Care Assessment Tool modified for use in NZ (WellCCAT-NZ) Score Sheet

Centre name: **Date of Assessment:**

			**Examples:** *"If children have health needs that we cannot meet, we shall refer parents to appropriate agencies, such as the child's doctor or Regional Public Health Service"* *"Nutrition education for parents is also provided through information that is sent home and at parent night events"* *"The facility shall schedule regular nutrition information sessions for parents"*

CE3	**Specifies a suitable timeframe for revising and updating the centre food and nutrition policy.**	0	Not mentioned, or the stated date for revision has passed or is blank.
		1	**Either of the following:**<br>• Vague and/or suggested.<br>• Mentions revisions (e.g., changes, additions, or recommendations) to policies or program but does not refer specifically to the health/wellness policies or programs.<br>• Has a specific review date (e.g. review by February 2016) but does not specify the process for review
		2	Identifies a plan for revising the health, wellness, or nutrition policies or programs.<br>**Examples:** *"An annual review conducted by senior management and a registered dietitian will make recommendations to ensure that the policy is fulfilling centre vision and goals"* *"Parents are requested to comment on this policy and our menus annually. Their comments are reviewed by management and the policy updated accordingly"* *"Health policy and program improvements are made as a result of the Parent Committee;s annual assessment findings"*

Supplementary S3: Infographic of results developed as feedback to all regional Early Learning Services.

The Nourishing Hawke's Bay: He wairua tō te kai surveyed early learning services across Hawke's Bay in term four 2020 about the healthiness of food environment policies and practices.

Aiming to improve food environments for children in Hawke's Bay

60 registered early learning services across Hawke's Bay

Response rate of **38%** of invited centres

90% Centre Manager / Director / Head Teacher / Kaiwhakahaere

6% Teachers

4% Parents / whānau / volunteers

Policies* on food and beverages

97% have food and / or nutrition policies

87% have drinks / beverages policies

Only **1 of 14** policies provided was "water-only"

4 policies mention water as "preferred or recommended"

*a written policy / procedure or guideline, for staff and / or parents

Provision of kai

1/3 provide **all** meals/snacks → serve food about **four** times per day

Almost **1/2** provide **some** meals/snacks → serve food about **twice** per day

1/4 provide **no** meals/snacks

*Of centres providing **all** or **some** kai (n = 42), they provided...*

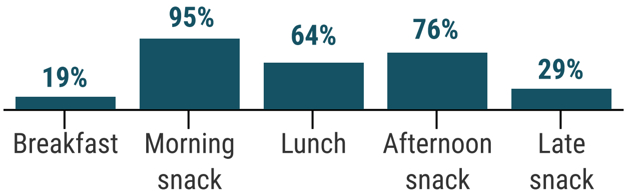

*Of centres providing **all** kai, at least a **morning snack** and **lunch** was provided*

Recommendations - what is needed?

A **water-only** policy

Preferred / recommended is not strong enough

Professional learning and development for teachers

More participation in food and nutrition programmes

Greater communication about policies with whānau

National Science Challenges
The University of Auckland
Medical and Health Sciences, School of Population Health
EIT Eastern Institute of Technology Te Aho a Māui
Hawke's Bay District Health Board Whakawāteatia
cure kids
This project was made possible with funding from the National Science Challenge and Cure Kids

Programmes and barriers

Many centres **participate** in Food and /or Nutrition Programmes (62%)

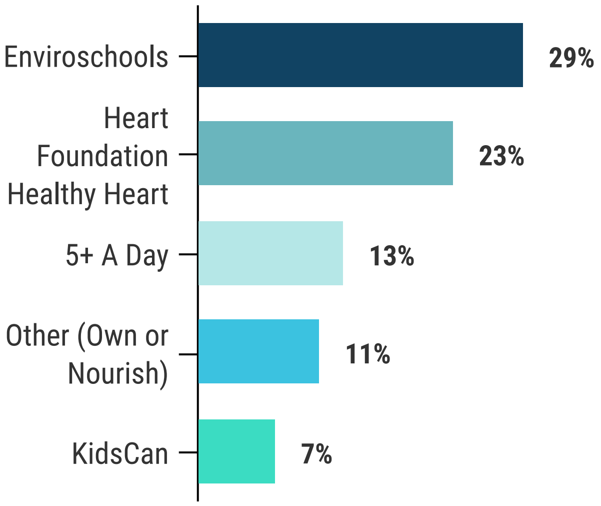

Around half of centres **report barriers** to providing or promoting healthy food to children / tamariki (58%)

The most common barriers are:

- Lack of support from parents / whānau (32%)
- Concerns about food-related choking (30%)
- Concerns about food intolerances or allergies (25%)
- Insufficient funds (21%)

Culture around healthy eating

Most centres **have a strong culture** around healthy eating

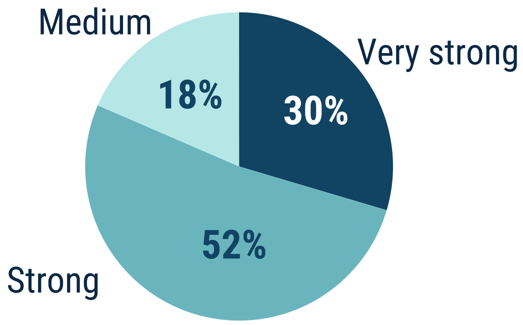

Very strong = policies in place, strong healthy food practices, staff / parents strongly support healthy food kaupapa
Strong
Medium = some policies and practices support healthy food, mixed support from staff / parents for healthy food kaupapa

We asked centres how much they agreed with the following statements

There was strong support for...

- **86%** a **strong collective vision** around hauora / health between centre **management** and **staff**
- **84%** the centre **highly prioritises nutrition** and healthy eating
- **75%** staff consistently act as **role models** for healthy eating
- **70%** frequent **communication with parents** and whānau about nutrition and healthy eating

There was moderate support for...

- **60%** a **strong collective vision** around hauora / health between centre **management** and **parents / whānau**
- **44%** the provision of **regular nutrition training** for all **staff**

*aggregate of 'strongly agree' and 'agree' responses

Special occasions and fundraising

Food and drinks for **special occasions** are **mostly healthy** when provided by the centre

Almost **3/4** of centres **provide** food / beverages for special occasions

The ratio of green to red items is...

3 : 1

green : red

Green items ('everyday' healthy items like fruit, vegetables, plain milk, cereals, bread, etc.)

Red items ('occasional' unhealthy items - muffins, pizza, sausage rolls, cake, flavoured drinks & chips)

4/5 allow children to bring food / beverages for special occasions **from home**

The ratio of green to red items is...

1 : 1

green : red

*Food for special occasions brought from **home is less healthy** than food provided by the centre*

Few centres **use food and beverages in fundraising**, and only infrequently.

When food is used in fundraising, it's generally not healthy

1/5 centres use food and / or beverages in fundraising

The ratio of green to red items used in fundraising

2 : 3

green : red

How frequent do centres fundraise?

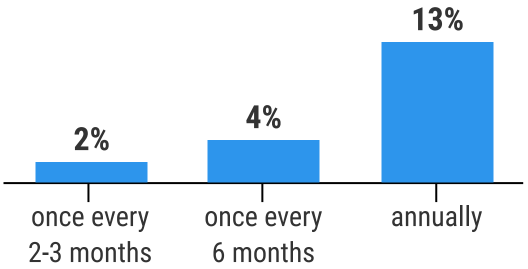

What might enable healthier food environments in early learning services?

Healthy eating in curriculum

Recipe ideas

More flexible MoE guidelines

Funding to provide healthier kai

Parent education

Healthy Heart programme

More buy-in from staff

Vege gardens

"We monitor and **communicate** verbally & face to face / newsletters with matua.

We now have **cake only for birthday** celebrations (used to be multiple pizzas).

We worked really hard for 2 years changing culture and aligned it to **physical active play** with Sports Hawke's Bay but it's now established.

Lots of communication within staff team & with parents, also a **milestone in our strategic plan under sustainability**."

Early learning services policy analysis

This policy analysis used the ***shortened WellCCAT-NZ tool (14 centres)****

Average **comprehensiveness** score **5/10**

Average **strength** score **1.2/10**

Most policies did not use strong prescriptive wording, rather focusing on encouragement and recommendations

Many centres **did not** separate food safety / hygiene guidelines from nutrition guidelines.

Most centres **did not** communicate their policy with parents and caregivers.

Many policies mentioned that **staff consistently role modelled** healthy eating

*WellCCAT = Wellness Child Care Assessment Tool

Menu analysis

2/3 of the food on the daily food menus in centres is healthy*

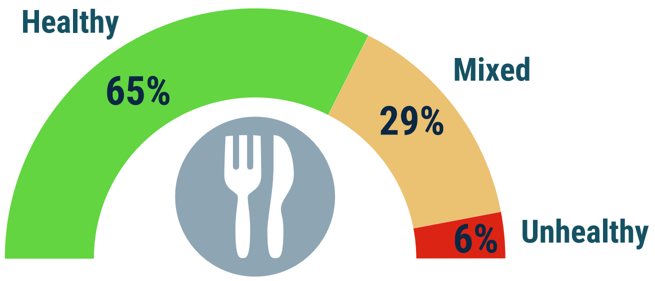

The proportion of unhealthy items varied across centres from 0% to 36%

*According to 2020 NZ Ministry of Healthy Food and Drink Guidance for Early Learning Services (18 menus analysed)

Summary of key findings

- **Almost all** centres report having **policies on food and beverages**
- **Most** centres **provide kai**
- **Many** centres **participate** in Food and /or Nutrition Programmes
- **Around half** centres **report barriers** to providing or promoting healthy food to children/tamariki
- Food and drinks for **special occasions** are **mostly healthy** when provided by the centre
- When food is used in **fundraising,** it's generally **not healthy**

***Most** centres have a **strong culture** around healthy eating*

Full results available at: https://www.eit.ac.nz/research-innovation/nourishing-hawkes-bay-he-wairua-to-te-kai/
For more information, please contact: nourishinghb@eit.ac.nz
Nourishing HB: He wairua tō te kai has ethic approval from the EIT Research Ethics and Approvals committee (Ref 20/03).
Date of report: Nov 2021
